# Structural and bioactive studies of terpenes and cyclopeptides from the Genus *Rubia*

**DOI:** 10.1186/1752-153X-7-81

**Published:** 2013-05-04

**Authors:** Kuo Xu, Penglong Wang, Bin Yuan, Yatao Cheng, Qiang Li, Haimin Lei

**Affiliations:** 1 School of Chinese Pharmacy, Beijing University of Chinese Medicine, Beijing, 100102, China

**Keywords:** Genus *Rubia*, Phytochemicals, Terpenes, Cyclopeptides, Bioactivities

## Abstract

Genus *Rubia* fell into about 70 species distributed widely around the world, a total of 36 species and 2 varieties were reported from China. The extracts and phytochemicals of *Rubia* plants had drawn considerable attention due to their potent bioactivities. As the two major ingredients from these plants, pentacyclic triterpenes and cyclopeptides were becoming a hot topic over the past twenty years for their remarkable anticancer, antioxidant and other effects. This paper compiled all 65 terpenes and 44 cyclopeptides with their distributions, physiological activities and melting points (or optical rotations) as reported in 85 references; besides, structure-activity relationships of these derivatives were briefly discussed. The information involved in this paper was expected to be meaningful for the further studies of the Genus *Rubia*.

## Review

### Introduction

Genus *Rubia* that belongs to the *Rubiaceae* is one kind of perennial herbs; it falls into about 70 species distributed widely around the world, such as Western Europe, Northern Europe, Mediterranean Coast, Temperate Asia, Africa, Himalaya, as well as the regions from Mexico to Tropical America. A total of 36 species and 2 varieties have been reported from China [1]. *Rubia* species being one of the earliest plant resources possessed important commercial and medicinal values. Commercially, they were used as natural dye-stuffs in old days and improved commodity circulation; medically, these species being used as drugs were first recorded in the world famous pharmacy book of China, *Divine Famer*'*s Materia Medica*, which has over 2000 years history [2]. According to many medical books, the roots of *Rubia* plants being reputed for their satisfactory efficacy were wildly used for the treatment of cancers, tuberculosis, rheumatism, hematemesis, metrorrhagia, epistaxis, contusion and menoxenia in the Chinese traditional medicine [3,4]. Besides, Indian folk medicine also comprised numerous prescriptions involved in Genus *Rubia* for healing wounds, inflammation, skin infections, and so on [5].

Within the last few decades, the extracts and phytochemicals of *Rubia* plants have drawn much attention due to their potent bioactivities. Studies on these plants led to the isolation of a series of bioactive ingredients including anthraquinones, naphthoquinones, terpenes, cyclopeptides and other constituents. Nine years ago, R. Singh *etc*. summarized a total of 33 terpenes and 19 cyclopeptides identified from 7 *Rubia* species [6]. Since then, researches on phytochemicals of Genus *Rubia* have achieved a lot and the lack of a comprehensive and lately review on this subject prompted us to gather much more new information. The previous review of us has introduced 148 anthraquinones and naphthoquinones together with their structure-activity relationships; herein we continue a general presentation of *Rubia* terpenes and cyclopeptides that exhibited remarkable anticancer, antioxidant and other effects. The present paper is dedicated to summarizing and updating a total of 65 terpenes and 44 cyclopeptides whose structures, distributions and properties are listed in the following tables and figures; furthermore, structure-activity relationships of these derivatives are briefly discussed.

### Terpenes and their glycosides isolated from the Genus *Rubia*

Terpenes were not generally considered as major effective ingredients existed in medicinal *Rubia* plants, but they were also the earliest phytochemicals isolated from Genus *Rubia*. To our knowledge, people have got 65 terpene derivatives including 5 monoterpenes and 60 pentacyclic triterpenes. Compared with the similar work in 2004, this paper supplemented 32 new terpenes; plant sources of several compounds were also extended. What’s more, we modified some previous information; for instance, β-sitosterol, daucosterol and stigmasterol should be classified under the steroids rather than the terpenes; three pentacyclic triterpenes named ‘Rubiprasins A-C’ were mistaken as ‘Rubiprassins A-C’ and Rubiprasin C’s structure was also wrongly described. All of the 65 terpenes and their glycosidic derivatives isolated from 8 *Rubia* species are listed in Table 1 together with melting points (or optical rotations); the structures of these compounds are described in Additional file 1: Figure S1.

**Table 1:** **Structures of terpenes** (**1**–**65**) **and cyclopeptides** (**66**–**109**) **isolated from the Genus *Rubia***

**NO.**	**Structures’ name**	**Mp/°C or [α]/°**	**Resources &References**
**1**	6-methoxygeniposidic acid	—	*R*. *cordifolia*[7]
**2**	Asperuloside	173-175 [8]	*R*. *tinctorum*[8]
		130–132 [33]	*R*. *peregrina*[9]
**3**	Asperulosidic acid	[α]_D_ +21 [33]	*R*. *peregrina*[9]
**4**	Deacetylasperulosidic acid	[α]_D_ +21 [33]	*R*. *peregrina*[9]
**5**	3R,3aS,4R,6aR-3,4,6-tris (hydroxymethyl)-3,3a,4,6a-tetrahydro-2H-cyclopenta[b] furan-2-one	${\left[\alpha \right]}_{D}^{22}+4$ [25]	*R*. *cordifolia*[25]
**6**	Rubiacoumaric acid	215/ [α]_D_ +24.5 [10]	*R*. *cordifolia*[10]
**7**	Rubiafolic acid	286-287/ [α]_D_ +58.8 [10]	*R*. *cordifolia*[10]
			*R*. *schumanniana*[20]
**8**	Zamanic acid	${\left[\alpha \right]}_{D}^{27}+26$ [35]	*R*. *schumanniana*[20]
**9**	Maslinic acid	269-271 [31]	*R*. *schumanniana*[20]
			*R*. *yunnanensis*[22]
**10**	Spathodic acid	280 [37]	*R*. *yunnanensis*[22]
**11**	Ursolic acid	272-273 [32]	*R*. *schumanniana*[20]
			*R*. *yunnanensis*[22]
**12**	Oleanolic acid	277.6-280.1 [31]	*R*. *schumanniana*[20]
		281–282 [32]	*R*. *cordifolia*[26]
		280–282 [34]	*R*. *ustulata*[27]
**13**	Karachic acid	260-261/ [α]_D_ +79 [29]	*R*. *schumanniana*[20]
**14**	3-O-acetyloleanolic acid	265-266 [34]	*R*. *ustulata*[27]
**15**	Oleanolic aldehyde acetate	216-218 [36]	*R*. *cordifolia*[7]
**16**	Rubiarbonol A	260-263/ [α]_D_ +36.5 [11]	*R*. *cordifolia*[11,24]
		260–262 [13]	*R*. *yunnanensis*[13,15,16,18,21,22]
		289–290 [16,18]	
**17**	Rubiarbonol B	272-273/ [α]_D_ +30.3 [11]	*R*. *oncotricha*[11]
			*R*. *yunnanensis*[15]
			*R*. *cordifolia*[24]
			*R*. *ustulata*[27]
**18**	Rubiarbonol C	211-213/ [α]_D_ –4.3 [11]	*R*. *oncotricha*[11]
**19**	Rubiarbonol D	218-220/ [α]_D_ +6.1 [11]	*R*. *oncotricha*[11]
			*R*. *akane*[28]
**20**	Rubiarbonol E	290/ [α]_D_ +15.4 [11]	*R*. *oncotricha*[11]
**21**	Rubiarbonol F	280/ [α]_D_ +33.3 [11]	*R*. *oncotricha*[11]
			*R*. *yunnanensis*[15]
			*R*. *yunnanensis*[21,22]
**22**	Rubiarbonol G	170-173 [13]	*R*. *yunnanensis*[13,15,16,18,22,30]
		174–176 [16,18]	
**23**	Rubiarbonol K	—	*R*. *schumanniana*[20]
			*R*. *yunnanensis*[22,30]
**24**	Rubiarbonol L	—	*R*. *yunnanensis*[22,30]
**25**	Rubiarbonone A	153-155 [13]	*R*. *yunnanensis*[13,15,16,18,22,30]
		160–162 [16,18]	
**26**	Rubiarbonone B	155-157 [14]	*R*. *yunnanensis*[14]–[16,18,21,22]
		255–256 [16,18]	
**27**	Rubiarbonone C	98-100 [14]	*R*. *yunnanensis*[14,21,22]
**28**	Rubiarbonone D	231-232/ [α]_D_ +94.4 [15]	*R*. *yunnanensis*[15]
**29**	Rubiarbonone E	258-259/ [α]_D_ +233.4[15]	*R*. *yunnanensis*[15,22]
**30**	Rubiarbonone F	253-254/ [α]_D_ +26.4 [15]	*R*. *yunnanensis*[15]
**31**	Rubiarboside A	—	*R*. *yunnanensis*[15,21,22,30]
**32**	Rubiarboside B	—	*R*. *yunnanensis*[21,30]
**33**	Rubiarboside C	—	*R*. *yunnanensis*[21,22,30]
**34**	Rubiarboside F	294-295/ [α]_D_ +98.1 [15]	*R*. *yunnanensis*[15]
**35**	Rubiarboside G	>290/ [α]_D_ +56.4 [15]	*R*. *yunnanensis*[15,22]
**36**	Rubiprasin A	>300/ [α]_D_ +12.8 [12]	*R*. *cordifolia*[12]
			*R*. *ustulata*[27]
			*R*. *akane*[28]
**37**	Rubiprasin B	277-280 [12]	*R*. *cordifolia*[12]
			*R*. *ustulata*[27]
			*R*. *akane*[28]
**38**	Rubiprasin C	171-173 [12]	*R*. *cordifolia*[12]
**39**	Rubiatriol	252-256 [17]	*R*. *cordifolia*[17]
**40**	Rubianol-a	${\left[\alpha \right]}_{D}^{25}+10.0$ [19]	*R*. *yunnanensis*[19,21]
**41**	Rubianol-b	${\left[\alpha \right]}_{D}^{25}+16.8$ [19]	*R*. *yunnanensis*[19,21]
**42**	Rubianol-c	${\left[\alpha \right]}_{D}^{25}+36.4$ [19]	*R*. *yunnanensis*[19,21,22]
**43**	Rubianol-d	${\left[\alpha \right]}_{D}^{25}+63.6$ [19]	*R*. *yunnanensis*[19,21,22]
**44**	Rubianol-e	${\left[\alpha \right]}_{D}^{25}+18.1$ [19]	*R*. *yunnanensis*[19,21,22]
**45**	Rubianol-g	${\left[\alpha \right]}_{D}^{25}+206.1$ [21]	*R*. *yunnanensis*[21]
**46**	Rubianoside I	${\left[\alpha \right]}_{D}^{25}+10.9$ [19]	*R*. *yunnanensis*[19,21,22]
**47**	Rubianoside II	${\left[\alpha \right]}_{D}^{25}+2.2$ [21]	*R*. *yunnanensis*[21]
**48**	Rubianoside III	${\left[\alpha \right]}_{D}^{25}+3.5$ [21]	*R*. *yunnanensis*[21]
**49**	Rubianoside IV	${\left[\alpha \right]}_{D}^{25}+90.5$ [21]	*R*. *yunnanensis*[21]
**50**	3β,6α-dihydroxy-urs-14-en-12-one	${\left[\alpha \right]}_{D}^{20}–10.7$ [20]	*R*. *schumanniana*[20]
**51**	3β-hydroxy-urs-30-*p*-*Z*-hydroxycinnamoyl-12-en-28-oic-acid	${\left[\alpha \right]}_{D}^{16}+6.5$ [20]	*R*. *schumanniana*[20]
**52**	3β-hydroxy-olean-30-*p*-*E*-hydroxycinnamoyl-12-en-28-oic-acid	${\left[\alpha \right]}_{D}^{16}+13.1$ [20]	*R*. *schumanniana*[20]
**53**	Rubiarbonol A 7-acetate	${\left[\alpha \right]}_{D}^{23}+1.7$ [22]	*R*. *yunnanensis*[22]
**54**	Rubiyunnanol A	${\left[\alpha \right]}_{D}^{16}+23.2$ [22]	*R*. *yunnanensis*[22]
**55**	Rubiyunnanol B	$247-248/{\left[\alpha \right]}_{D}^{16}+25.8$ [22]	*R*. *yunnanensis*[22]
**56**	19,28-Didehydroxyrubiarbonol A	${\left[\alpha \right]}_{D}^{16}+39.7$ [22]	*R*. *yunnanensis*[22]
**57**	Rubiyunnanol C	${\left[\alpha \right]}_{D}^{18}–28.9$ [22]	*R*. *yunnanensis*[22]
**58**	Rubiarbonone E 19-acetate	$259-260/{\left[\alpha \right]}_{D}^{23}–4.6$ [22]	*R*. *yunnanensis*[22]
**59**	2-Hydroxyrubiarbonone E	${\left[\alpha \right]}_{D}^{23}+19.7$ [22]	*R*. *yunnanensis*[22]
**60**	Rubianol-e3-O-(6’-O-acetyl)-β-D-Gluc	${\left[\alpha \right]}_{D}^{25}–25.2$ [22]	*R*. *yunnanensis*[22]
**61**	2α-Acetoxy-28-acetylrubiarboside G	${\left[\alpha \right]}_{D}^{16}–27.9$ [22]	*R*. *yunnanensis*[22]
**62**	Rubiarboside G 28-acetate	${\left[\alpha \right]}_{D}^{16}–21.7$ [22]	*R*. *yunnanensis*[22]
**63**	3-β-friedelinol	281-283 [23]	*R*. *cordifolia*[23]
**64**	Rubiarboside G 28-al	${\left[\alpha \right]}_{D}^{16}–41.7$ [22]	*R*. *yunnanensis*[22]
**65**	Rubiarbonol A 3-O-β-D-glucopyranosyl- (1–2)- β-D-Gluc	${\left[\alpha \right]}_{D}^{16}–9.0$ –9.0 [22]	*R*. *yunnanensis*[22]

### Monoterpenes

Monoterpenes hold a relatively small proportion among *Rubia* terpenes. Up to 2012, researchers have identified 5 monoterpenes (1–5) distributed in 3 species including *R*. *cordifolia*, *R*. *tinctorum* and *R*. *peregrina*[7]–[9]; and it’s just the continuous studies of the ethyl acetate fraction of *R*. *cordifolia* that resulted in the isolation of the monoterpenoid (5). More detailed information of monoterpenes is involved in the following Table and Figure.

### Pentacyclic triterpenes

Most of *Rubia* pentacyclic triterpenes came from three species including *R*. *schumanniana*, *R*. *peregrina*, and *R*. *yunnanensis*, especially the latter. *R*. *yunnanensis* is known as “Xiao-Hong-Shen”, which is endemic to China and has been served as an alternative for *R*. *cordifolia*; it has a long history of medicinal application in China. The aforementioned three *Rubia* species were rich sources for pentacyclic triterpenes. No report could be available on triterpene derivatives of Genus *Rubia* until 1981, when S. K. Talapatra *etc*. got Rubiacoumaric acid (6) and Rubiafolic acid (7) from *R*. *cordifolia*[10]. Several years after the discovery of the first two triterpenes, rubiprasins A-C and rubiarbonols A-F were identified from the same species [11,12]. The subsequent reported pentacyclic triterpenes were mainly designated under a series of names such as rubiarbonol, rubiarbonone, rubiarboside, rubiprasin and rubianol. It seemed that compounds named rubiarbonones, rubiarbosides, rubianols and rubianosides mainly existed in *R*. *yunnanensis*[13]–[22]. Friedelinol-type triterpene (63) that belonged to arborinane-type derivatives was previously isolated from the family *Rubiaceae*, but it’s the first report for the isolation from the Genus *Rubia*[23]. Further phytochemical studies of Genus *Rubia* have resulted in identification of another 16 new triterpenes [24]–[37].

Except for compiling and summarizing the latest information, in the process of literature searching, we deem that some previous information is not credible. For instance, melting points of Rubiarbonone B (26) which roots in two different references should not be deviated too much [14,16,18]; it’s also doubted that the structure of Rubiarboside F (34) was describe as IV’s (49) [15,21]. More detailed information of *Rubia* triterpenes is listed in the following Table and Figure, Mp refers to Melting point; [α] means to the Specific Rotation of the compounds (due to different test conditions, the data may be various).

### Cyclopeptides isolated from the Genus *Rubia*

Studies on plant cyclopeptides have drawn considerable attention for their distinctive bicyclic structural features and significant antitumor activities. On the basis of the chemical skeletons and distributions in plants, N. H. Tan *etc*. proposed the systematic structural classification of plant cyclopeptides which were divided into two classes, five subclasses, and eight types [38]. Followed by the rule, *Rubia* cyclopeptides should be classified under the *Rubiaceae*-type cyclopeptides formed with one α-D-alanine, one α-L-alanine, three *N*-methyl-α-L-tyrosines and one other proteinogenic α-L-amino acid [39]. So far, people have got 44 cyclopeptide derivatives from the Genus *Rubia*. Compared with terpene derivatives, *Rubia* cyclopeptides have narrower sources and only existed in three species containing *R*. *cordifolia*, *R*. *yunnanensis* and *R*. *akane*. In general, according to these cyclopeptides’ names, we may deduce their distributions; for instance, the cyclopeptides named Rubiyunnanins and RYs were reported from *R*. *yunnanensis* while the structures whose names were designated as RAs or others mainly came from *R*. *cordifolia*.

At the end of the twentieth century, cyclopeptides called RA-I (66) and RA-II (67) were isolated from chloroform and methanol extracts of *R*. *cordifolia* as the minor constituents [40]. It’s the first time to report that Genus *Rubia* contained cyclopeptides. Later, RAs III-VII (68, 70–74) were obtained from the methanol extract of *R*. *cordifolia* together with *R*. *akane*[41]–[44]. As far as we know, among the reported cyclopeptides, only RA-V (71) and RA-VII (74) existed in *R*. *akane*. Except for aglycone, further studies on the Genus *Rubia* have also confirmed many cyclopeptide glycosides; these known glucosides were just single substituted glucopyranoside derivatives. For example, RA-XII (79), RA-XIII (80) and RA-XIV (81) were discovered in higher plants for the first time [45]; RA-XV (82) and RA-XVI (83) that belonged to glucosides were identified from *R*. *cordifolia*[46]; in addition, J. T. Fan *etc*. reported that Rubiyunnanins B (93), F (97), G (98), H (99), and RYs I-III (100–102) also contained glycosyl [47]–[58]. Beyond that mentioned skeleton, uncommonly, chloroform-soluble portion of methanol extracts from the roots of *R*. *cordifolia* gave a dimer named RA-dimer A (103), in which two molecules of deoxybouvardin were linked together via an ether linkage [59]; however, it’s a pity for us that chemical constructions of RA-700 and RC-18 haven’t been available [60,61]. Just in last year, Y. Hitotsuyanagi *etc*. got a series of special cyclopeptides from the Genus *Rubia*[62,63].

The complete list of cyclopeptides isolated from 3 *Rubia* species is summarized in Table 2 together with the physical properties; their structures are described in Additional file 2: Figure S2; herein Mp refers to Melting point, [α] means to the Specific Rotation (due to different test conditions, the data may be various), β-D-Gluc refers to β-D-glucopyranoside.

**Table 2:** **Structures of terpenes** (**1**–**65**) **and cyclopeptides** (**66**–**109**) **isolated from the Genus *Rubia***

**NO.**	**Derivatives’ names**	**Mp/°C or [α]/°**	**Distributions &References**
**66**	RA-I	284 [40]	*R*. *cordifolia*[40]
			*R*. *yunnanensis*[55]
**67**	RA-II	261 [40]	*R*. *cordifolia*[40]
**68**	RA-III	>300 [40]/ ${\left[\alpha \right]}_{D}^{28}–199$[41]	*R*. *cordifolia*[40,41,44]
**69**	RAI-III	209-211/ [α] _D_ –38.3 [48]	*R*. *cordifolia*[48]
**70**	RA-IV	247-255 [40]/ $\;{\left[\alpha \right]}_{D}^{28}–126$[41]	*R*. *cordifolia*[40,41,44]
**71**	RA-V	>300/ $\;{\left[\alpha \right]}_{D}^{21}–225$[41]	*R*. *cordifolia*[41,44]
			*R*. *akane*[41]
			*R*. *yunnanensis*[55,56]
**72**	RA-VI	219-220/ [α] _D_ –118.6 [42]	*R*. *cordifolia*[42]
**73**	RAI-VI	200-202/ [α] _D_ –129.4 [48]	*R*. *cordifolia*[48]
**74**	RA-VII	>300/ ${\left[\alpha \right]}_{D}^{21}–229$[41]	*R*. *cordifolia*[41,44,50]
			*R*. *akane*[41]
**75**	RA-VIII	267-269/ [α] _D_ –159.5 [42]	*R*. *cordifolia*[42]
**76**	RA-IX	242-243/ $\;{\left[\alpha \right]}_{D}^{20}–158.1$[43]	*R*. *cordifolia*[43]
**77**	RA-X	254.5-255.5/ $\;{\left[\alpha \right]}_{D}^{20}–205.4$[43]	*R*. *cordifolia*[43]
**78**	RA-XI	255.5/ [α] _D_ –235.8 [45]	*R*. *cordifolia*[45]
**79**	RA-XII	252-255/ [α] _D_ –270.0 [45]	*R*. *cordifolia*[45,49]
			*R*. *yunnanensis*[21,55]
**80**	RA-XIII	273-276/ [α] _D_ –109.3 [45]	*R*. *cordifolia*[45]
**81**	RA-XIV	264-267/ [α] _D_ –257.8 [45]	*R*. *cordifolia*[45]
**82**	RA-XV	218-220/ [α] _D_ –202.4 [46]	*R*. *cordifolia*[46]
**83**	RA-XVI	220/ [α] _D_ –179.7 [46]	*R*. *cordifolia*[46]
**84**	RA-XVII	${\left[\alpha \right]}_{D}^{24}–194$ [47]	*R*. *cordifolia*[47]
**85**	RA- XVIII	${\left[\alpha \right]}_{D}^{25}–222$ [51]	*R*. *cordifolia*[51]
**86**	RA- XIX	${\left[\alpha \right]}_{D}^{26}–224.4$ [52]	*R*. *cordifolia*[52]
**87**	RA- XX	${\left[\alpha \right]}_{D}^{26}–218.4$ [52]	*R*. *cordifolia*[52]
**88**	RA- XXI	>300/ ${\left[\alpha \right]}_{D}^{26}–230.1$[52]	*R*. *cordifolia*[52]
**89**	RA- XXII	${\left[\alpha \right]}_{D}^{26}–186.7$ [52]	*R*. *cordifolia*[52]
**90**	RA- XXIII	254-256/ $\;{\left[\alpha \right]}_{D}^{26}–184.2$[53]	*R*. *cordifolia*[53]
**91**	RA- XXIV	258-261/ $\;{\left[\alpha \right]}_{D}^{26}–168.5$[53]	*R*. *cordifolia*[53,63]
			*R*. *yunnanensis*[55]
**92**	Rubiyunnanin A	${\left[\alpha \right]}_{D}^{28}–115.8$ [54]	*R*. *yunnanensis*[54]
**93**	Rubiyunnanin B	${\left[\alpha \right]}_{D}^{24}–205.8$ [54]	*R*. *yunnanensis*[54]
**94**	Rubiyunnanin C	253-254/ $\;{\left[\alpha \right]}_{D}^{27}–221.0$[55]	*R*. *yunnanensis*[55]
**95**	Rubiyunnanin D	${\left[\alpha \right]}_{D}^{23}–149.7$ [55]	*R*. *yunnanensis*[55]
**96**	Rubiyunnanin E	${\left[\alpha \right]}_{D}^{23}–124.5$ [55]	*R*. *yunnanensis*[55]
**97**	Rubiyunnanin F	${\left[\alpha \right]}_{D}^{25}–182.5$ [55]	*R*. *yunnanensis*[55]
**98**	Rubiyunnanin G	${\left[\alpha \right]}_{D}^{28}–127.3$ [55]	*R*. *yunnanensis*[55]
**99**	Rubiyunnanin H	${\left[\alpha \right]}_{D}^{27}–244.0$ [55]	*R*. *yunnanensis*[55]
**100**	RY-I	226-228/ $\;{\left[\alpha \right]}_{D}^{25}–267$[56]	*R*. *yunnanensis*[56]
**101**	RY-II	—	*R*. *yunnanensis*[55,57]
**102**	RY-III	—	*R*. *yunnanensis*[6,58]
**103**	RA-dimer A	${\left[\alpha \right]}_{D}^{25}–247$ [59]	*R*. *cordifolia*[59]
**104**	RA-700	—	*R*. *cordifolia*[6,60]
**105**	RC-18	—	*R*. *cordifolia*[6,61]
**106**	Allo-RA-V	${\left[\alpha \right]}_{D}^{25}–234$ [62]	*R*. *cordifolia*[62]
**107**	Neo-RA-V	${\left[\alpha \right]}_{D}^{25}–290$ [62]	*R*. *cordifolia*[62]
**108**	O-seco-RA-V	${\left[\alpha \right]}_{D}^{25}–82$ [62]	*R*. *cordifolia*[62]
**109**	O-seco-RA-XXIV	${\left[\alpha \right]}_{D}^{25}–62$ [63]	*R*. *cordifolia*[63]

### Biological activities of *Rubia* terpenes and *Rubia* cyclopeptides

Various crude fractions and purified compounds from the Genus *Rubia* exhibited a relatively wide range of biological activities [64]–[69]. Numerous experimental data verified that pentacyclic triterpenes possessed potent advantage on the aspects of antitumor, antiinflammatory, protecting liver, anti-HIV effects [70], while the most significant advantage of plant cyclopeptides was their underlying mechanisms of antitumor effects. As the two major ingredients existed in those *Rubia* plants, pentacyclic triterpenes and cyclopeptides were becoming a hot topic over the past twenty years for their remarkable effects. The following contents aims to offer the detailed statements of pharmacological activities of *Rubia* terpenes and cyclopeptides, especially their anticancer and antioxidant effects. Finally, all the bioactive terpenes and cyclopeptides together with the sketchy mechanisms are collected in Table 3.

**Table 3:** **The biological activities of terpenes and cyclopeptides from the Genus *Rubia***

**Parts**	**Biological activities**	**Mechanisms of action**	**Compounds’ code names**
**4**.**1**	Anticancer effect	Cytotoxicity and inhibitory on cell lines	**8**, **9**, **11**, **15**, **16**, **21**, **22**, **27**, **42**, **50**, **52**, **54**, **55**, **58**, **62**, **66**–**71**, **74**, **77**–**81**, **84**–**102**, **104**, **105**, **109**
		Inducing cell apoptosis.	**9**, **11**, **12**
		Antiangiogenic	**71**, **74**
**4**.**2**	Antioxidant effect	NO production	**16**, **21**, **27**, **42**, **43**, **44**, **66**, **71**, **79**, **91**, **93**–**99**, **101**
**4**.**3**	Other effects	Antiplatelet aggregation	**16**, **17**, **25**
		Antimicrobial	**11**, **12**, **22**
		Antiobesity	**11**, **12**
		Antidiabetic	**9**, **11**, **12**
		Anti-HIV	**9**, **11**

**Notice**: Compounds’ Code Names are involved in these derivatives listed in Table 1 and Table 2.

### Anticancer effects of *Rubia* terpenes and *Rubia* cyclopeptides

#### Cytotoxicity and inhibitory on cell lines

Rubiarbonols A (16) and F (21), rubiarbonone C (27), and rubianol-c (42) exhibited cytotoxic effects in the MTT assay and they could also inhibit NO production [19]. Compounds 7–9, 11–13, 23 and 50–52 had been evaluated for cytotoxicity against three human cancer cell lines including Hela, BGC-823 and A549; then compounds 8–9, 11, 50 and 52 showed cytotoxicity with the IC_50_ values of 10.75~18.87 μg/ml [20]. Compounds 22, 54, 55, 58 and 62 had inhibition on A549, Hela and SMMC-7721; however, the data were not very satisfactory [22]. Rubiarbonol A (16) was reported to have relatively strong cytotoxicity against HT-29; compared rubiarbonols A and B, introduction of the hydroxyl group on C-28 seemed to enhance cytotoxicity, especially on HT-29. Besides, compound 15 exhibited potent bioactivities against A549, SK-OV-3, SK-MEL-2, MES-SA and HCT-15 [36].

Different from pentacyclic triterpenes’ wide bioactivities, the effects of *Rubia* cyclopeptides mainly focused on inhibitory against tumors; and it’s available to identify bioactive cyclopeptides. An efficient isolation method for antitumor cyclopeptides from Genus *Rubia* had been established by following the activity against murine tumor P388 leukemia that was successfully used to purify RAs I-IV (66–70) [40]. In a similar way, H. Itokawa *etc*. [41] found that the methanolic extract prepared from roots of *R*. *cordifolia* had a significant antitumor activity against Sarcoma 180 ascites and P388 leukemia in mice, from which RAs-III, -IV, -V (71) and -VII (74) were obtained. Recent years, monoclonal antibodies against RA-VII (74) had been also generated for screening of antitumor cyclopeptides. T. Hasuda *etc*. proved that they were useful for checking of the RA series compounds in the roots of *R*. *cordifolia* and *R*. *akane*[71].

Various data exhibited that different structural residues of *Rubia* cyclopeptides seemed to be closely related with their anticancer effects. Studies on the effect of RA-XVII (84) on cytotoxicity and conformation showed that although the structure exhibited little effect on the conformation of the molecule, it might decrease the activity as the side chain of residue 1 grew longer [47]. On the basis of several cyclopeptide derivatives, the typical type II β-turn structure and the aromatic side chain of Tyr-3 over this turn were also considered to play a very important role in its antitumor activities [42]. RA-X (77) containing a glutamic acid at residue 2 possessed strong antitumour effect on P388 while RA-IX (76) with conformation restricted to a type II β-turn at residues 2 and 3 showed almost no antitumour activities [43]. RAs XI-XIV (78–81) had potent antitumor activity against P388, especially the compound 79 whose residue 2 was methyl. The conformation of *cis* N-methyl amide bond between residues 2 and 3 also played an important role in the antitumour activities of RAs [50]. RA-XVIII (85) was another natural peptide of the RA-series in which the benzene ring of Tyr-6 was hydroxylated and its cytotoxicity against P-388 was 0.012 μg/ml [51]. RA-XIX, -XX, -XXI and -XXII (86–89) had been evaluated for their cytotoxicity against P388, with RA-VII (74) as reference; for the compounds having a methoxyl group in Tyr-6, the order of cytotoxicity was 74 > 87 > 86. Compound 89 possessing a hydroxyl group in its Thr-2 was less cytotoxic than 88. It seemed that the cytotoxicity decreased with increased in the length of the carbon side chain or introduction of a polar functionality at this location [52]. RA-XXIII (90) and RA-XXIV (91) exhibited just moderate cytotoxicity against P-388 with IC_50_ values of 0.16 and 0.48 mg/ml, respectively [53]. T. Koizumi *etc*. had ever applied RA-VII (74) for the treatment of tumors in mice; daily intraperitoneal injection of RA-VII (1.5 or 3 mg/kg/day) had no toxic effects on those animals, but significantly and dose dependently inhibited the growth of Lewis lung carcinoma cells previously inoculated into the mice [72].

Compared with the various sources and remarkable effects of RAs, cyclopeptides named after Rubiyunnanin only existed in *R*. *yunnanensis* and their efficacies on tumors were relatively weaker. Cytotoxicities of Rubiyunnanin A (92) and B (93) against the 11 cancer cell lines were measured by SRB assay, but only 93 possessed moderate cytotoxicities [54]. Rubiyunnanins C-H (94–99) and RA-V, RA-I, RA-XXIV, RA-XII, RY-II not only exhibited cytotoxicities against the same cell lines with IC_50_ values ranging from 0.001 to 56.24 μM, but also exerted inhibitions against NO production in LPS and IFN-c-induced RAW 264.7 murine macrophages with IC_50_ values ranging from 0.05 to 12.68 μM [55]. RYs I-III (100–102), RA-700 (104), and RC-18 (105) possessed only medium activities against P388 [56]–[58,60,61]. The newly identified compounds 106–108, and RA-V, VII were evaluated for their cytotoxic activities against HL-60 and HCT-116 while only the latter had potent cytotoxicities [62]. Moreover, compound 109 exhibited cytotoxic effects on HL-60 cells, but which was much weaker than those of RA-XXIV and RA-VII [63].

#### Inducing cell apoptosis

Researchers paid much attention on anticancer mechanisms of maslinic acid (9), ursolic acid (11) and oleanolic acid (12) over the past decade years. No obvious correlation could be observed between cytotoxicity and inhibitory activity of DNA relaxation and decatenation by DNA topoisomerases I and II [24]. Oleanolic acid had selective inhibitory activity against DNA topoisomerase II compared with DNA topoisomerase I but weak cytotoxicity against HT-29, MCF-7 and HepG2 [26]. Maslinic acid was able to induce caspase-dependent apoptosis in human colon-cancer cells via the intrinsic mitochondrial pathway [73,74]; it could also potentiate anti-tumor activities of TNF-α and inhibit pancreatic tumor growth and invasion by activating caspase-dependent apoptotic pathway and by suppressing NF-κB activation and its downstream gene expression [75]. Inhibition of Protein kinase C (PKC) that was related to the tumor development might lead to inhibition of cells growth and spreading of cancer cells, while maslinic acid acted as a PKC inhibitor. Ursolic acid potentiated TRAIL-induced apoptosis through activation of reactive oxygen species and JNK-mediated up-regulation of death receptors and down-regulation of decoy receptor 2 and cell survival proteins [76,77]; it was also considered as a novel blocker of STAT3 activation that might have a potential in prevention and treatment of multiple myeloma and other cancers [78]. Ursolic acid and oleanolic acid possessed markedly apoptotic effects on four cell lines via increasing DNA fragmentation, decreasing mitochondrial membrane potential, lowering Na^+^-K^+^-ATPase activity and elevating caspase-3 and caspase-8 activities; they could suppress cell adhesion and reduce the production of VEGF and ICAM-1 in these cell lines [79].

#### Antiangiogenic effect

Compared with the inhibition of *Rubia* cyclopeptides on cell lines, antiangiogenic effect of them were rarely involved. Sato and his coworkers demonstrated the antiangiogenic activity of RA-VII (74) on the proliferation, migration, stress fiber formation of BAEC and effects on mouse corneal angiogenesis [72]. Last year, G. G. L. Yue *etc*. discovered RA-V (71) also possessed the activity in HUVEC and HMEC-1 with changes in function of these endothelial cells. The underlying mechanisms of action involved the ERK1/2 signalling pathway; however, RA-V might regulate different signalling pathways in various endothelial cells [80].

### Antioxidant effects of *Rubia* terpenes and *Rubia* cyclopeptides

Free radical NO was reported to be implicated in lots of physiological and pathological processes including vasodilation, nonspecific host defense and chronic or acute inflammation. It was produced by the oxidation of L-arginine under NO synthase (NOS). In the family of NOS, iNOS was particularly involved in pathological aspects with overproduction of NO, which could be expressed in response to pro-inflammatory agents such as interleukin-1β, tumor necrosis factor-a and lipopolysaccharide (LPS) in various cell types such as macrophages, endothelial cells and smooth muscle cells [21].

The bioactivities of rubianols on NO production from LPS-activated macrophages had been examined. Rubianol-d (43) and rubianol-e (44) exhibited the inhibition without cytotoxicity in the MTT assay whose efficacy was equivalent to L-NMMA’s; rubianol-c (42), rubiarbonols A (16) and F (21), rubiarbonone C (27) showed both cytotoxic and inhibitory activities on NO production [19]. RA-V (71) and RA-XII (79) were demonstrated to possess potent effects on iNOS induction, and the suppression was closely related to their inhibitions of NO [21]. Inhibitory effects of Rubiyunnanin A-H (92–99) and RA-V, RA-I, RA-XXIV, RA-XII, RY-I were also evaluated; all of the compounds except 92 exhibited activity against NO production in LPS [54].

### Other effects of *Rubia* triterpenes

#### Antiplatelet aggregation effect

Among compounds 16, 17 and 25, Rubiarbonol B (17) exhibited the most potent inhibition against arachidonic acid-induced and collagen-induced platelet aggregation at 150 μM, while Rubiarbonol A (16) and Rubiarbonone A (25) promoted platelet aggregation at the high doses and possessed antiplatelet aggregation activity at the lower concentrations. This result might be consistent with a basic tenet of Traditional Chinese Medicine, in that variations of the dose of prescriptions induced either stimulatory or inhibitory effects [15].

#### Antimicrobial effects

Antibacterial activity against *Staphylococcus aureus* and antifungal activity against *Candida albicans* of triterpenes from *R*. *yunnanensis* were evaluated using the turbidimetric method; compound 22 exhibited inhibition on both of the two strains [22]. Besides, ursolic acid (11) and oleanolic acid (12) possessed fairly high antimicrobial activities but were weaker than clinical antimicrobial drugs’; however, both of them exhibited low toxicity and might be used for treatment of infections by Vancomycin-Resistant Enterococci [81].

#### Antiobesity effect

Excess visceral adiposity might predispose to chronic diseases like hypertension and type-II diabetes with a high risk for coronary artery disease. Adipose tissue secreted cytokines and oxidative stress played an important role in the chronic disease progression. Triterpene derivatives had abilities to regulate glucose and lipid metabolism. C. L. Melo *etc*. [31] verified that oleanolic acid (12) could ameliorate visceral adiposity and improve glucose tolerance in mice and had an antiobesity potential through modulation of carbohydrate and fat metabolism. Much attention was also focused on food that might be beneficial in preventing diet-induced body fat accumulation and possibly reducing the risk of diabetes and heart disease; then ursolic acid (11) was demonstrated to have potent bioactivities improving certain metabolic parameters associated with diets high in saturated fats and obesity [82].

#### Antidiabetic effect

As a natural and low toxic compound, maslinic acid (9) elicited excellent outcomes without inducing the side effects. It exhibited significant glucose-lowering and hypoinsulinemic effects in KK-A^y^ mice and might hold great promise as a natural therapeutic agent for treatment of type-II diabetes [83]. Furthermore, both ursolic acid (11) and oleanolic acid (12) markedly suppressed renal aldose reductase activity and enhanced the activity of glyoxalase I, which contributed to decrease renal AGEs formation and improve renal functions; therefore, supplement of oleanolic acid and ursolic acid or foods rich in these compounds might be helpful for the prevention or treatment of diabetic kidney diseases [84].

#### Anti-HIV effect

Two triterpenes, maslinic acid (9) and ursolic acid (11), were reported to possess inhibition on the human immunodeficiency virus (HIV-1) protease [85].

## Conclusions

To our knowledge, the reported *Rubia* terpenes distributed in 8 species and most of them derived from *R*. *yunnanensis*. The triterpenes were designated under a series of names including rubiarbonol, rubiarbonone, rubiarboside, rubiprasin and rubianol; among them, oleanane-type and fernane-type triterpenes accounted for overwhelming majority. *Rubia* cyclopeptides had narrower sources and existed in only 3 species including *R*. *cordifolia*, *R*. *yunnanensis* and *R*. *akane*. These terpenes and cyclopeptides, especially the latter, possessed excellent anticancer effects; maslinic acid, ursolic acid, oleanolic acid and several cyclopeptides containing RA-V and RA-VII drew relatively more attention than any other compound. From Table 3 and Additional file 1: Figure S1, it may be inferred that the *Rubia* triterpenes owning a free hydroxyl group on third carbon atom exhibited relatively strong inhibitory on cell lines; besides, fernane-type triterpenes possessed promising antioxidant effects. As for *Rubia* cyclopeptides, increase in the length of carbon side chain at residues 1, 2 and introduction of polar groups at residue 2 might decrease structure’s cytotoxicity; N-methyl amide bond between residues 2 and 3, together with aromatic side chain of residue 3 over β-turn played pivotal role in the antitumor activities. Beyond that, the orientation of one or both of the Tyr-5 and Tyr-6 phenyl rings was also essential to express activities, while structures possessing two rings formed between Tyr-5 and residue 6 via a phenolic oxygen linkage and a new carbon bond only possessed moderate cytotoxicities.

Up to now, phytochemical investigations of the Genus *Rubia* have resulted in identification of various chemical components including anthraquinones, naphthoquinones, terpenes and cyclopeptides. This paper dedicated to compiling all 65 terpenes derivatives and 44 cyclopeptides derivatives from 8 *Rubia* species. Together with the previous work, we have already finished summarizing a total of 257 compounds from 12 *Rubia* species with their bioactivities. However, taking into account the fact that 70 species belong to the Genus *Rubia* spreading around the world, only 17% of them have been phytochemically investigated, the Genus *Rubia* still remains to be a potential resource to research. Furthermore, as one kind of natural dyestuffs, resource exploration of Genus *Rubia* is also an asset to economic development and waits for its full exploitation.

## Competing interests

The authors declare that they have no competing interests.

## Authors’ contributions

KX, PW, HL have all been involved in drafting this review. BY, YC, and QL read and approved the final manuscript. All authors read and approved the final manuscript.

## Supplementary Material

Additional file 1: Figure S1 Chemical structures of *Rubia* terpenes 1–65. Click here for file

Additional file 2: Figure S2 Chemical structures of *Rubia* cyclopeptides 66–109. Click here for file
