# Supplementary material for: Structural and bioactive studies of terpenes and cyclopeptides from the Genus Rubia
Source: Chem Cent J. 2013 May 4;7:81. doi: 10.1186/1752-153X-7-81 (PMC3698108; doi:10.1186/1752-153X-7-81)
Supplement: Additional file 1: Figure S1 — Chemical structures of Rubia terpenes 1–65. [file 1752-153X-7-81-S1.doc]

**Figure 1**. Chemical structures of *Rubia* terpenes **1-65**
