# Supplementary material for: Structural and bioactive studies of terpenes and cyclopeptides from the Genus Rubia
Source: Chem Cent J. 2013 May 4;7:81. doi: 10.1186/1752-153X-7-81 (PMC3698108; doi:10.1186/1752-153X-7-81)
Supplement: Additional file 2: Figure S2 — Chemical structures of Rubia cyclopeptides 66–109. [file 1752-153X-7-81-S2.doc]

**Figure 2**. Chemical structures of *Rubia* cyclopeptides **66-109**
